# Inclusive Education Implementation in Oman and Indonesia: A Comparative Analysis Study

**DOI:** 10.12688/f1000research.171420.1

**Published:** 2025-12-05

**Authors:** Siti Masyithoh, Ahmed Ali Loukam, Asep Ediana Latip, Andayani Andayani, Mochammad Noviadi Nugroho, Nuraida Nuraida, Rika Sa'diyah, Ati Kusmawati, Mu'arif SAM

**Affiliations:** 1 PGMI, State Islamic University Syarif Hidayatullah Jakarta Teacher Training and Islamic Education Faculty, South Tangerang, Banten, Indonesia; 2 Faculty of Shariah Science, Sultan Qaboos University, Mina Al Fahl, Muscat Governorate, Oman; 3 PGMI, State Islamic University Syarif Hidayatullah Jakarta Teacher Training and Islamic Education Faculty, South Tangerang, Banten, Indonesia; 4 PGSD, Universitas Terbuka, Jakarta, Special Capital Region of Jakarta, Indonesia; 5 Tadris IPS, State Islamic University Syarif Hidayatullah Jakarta Teacher Training and Islamic Education Faculty, South Tangerang, Banten, Indonesia; 6 PIAUD, Universitas Islam Negeri Ar-Raniry Fakultas Tarbiyah Dan Keguruan, Banda Aceh, Aceh, Indonesia; 7 MPI Pasca Sarjana, Muhammadiyah University of Jakarta, Jakarta, Special Capital Region of Jakarta, Indonesia; 8 Kesejahteraan Sosial, Muhammadiyah University of Jakarta, Jakarta, Special Capital Region of Jakarta, Indonesia; 9 Manajemen Pendidikan, State Islamic University Syarif Hidayatullah Jakarta Teacher Training and Islamic Education Faculty, South Tangerang, Banten, Indonesia

**Keywords:** inclusive education, implementation, comparative study, Oman, Indonesia, UDL, IEP.

## Abstract

**Background:**

Research comparing inclusive education implementation in non-Western contexts, such as Indonesia and Oman, remains limited. Both countries share cultural similarities yet differ significantly in their education governance. Understanding these differences is crucial for improving inclusive education policies and practices.

**Methods:**

This study employed qualitative document analysis, reviewing 33 policy and implementation documents from Indonesia and Oman. The analysis focused on five dimensions: service models, teacher training, curriculum adaptation, community involvement, and cultural attitudes.

**Results and Conclusions:**

Findings reveal that Oman’s centralized system provides consistent policy coordination but limits community participation and school flexibility. Indonesia’s decentralized system allows greater local innovation and community involvement but leads to disparities in implementation quality. Both countries struggle with teacher training, curriculum adaptation, and cultural stigma against disabilities. Effective inclusive education requires a balanced approach combining adaptive centralization and equitable decentralization, ongoing teacher development, flexible curricula, active community engagement, and cultural transformation. These insights offer practical recommendations to strengthen inclusive education in diverse socio-political contexts.

## Introduction

Inclusive education is a global agenda that has gained increasing attention since the proclamation of The Salamanca Statement and Framework for Action ^1, 2^ and the United Nations Convention on the Rights of Persons with Disabilities. ^3^ Both documents emphasize the importance of an education system that can accommodate all learners without discrimination, regardless of their abilities, socio-cultural background, or disability status. Thus, inclusive education is not merely an additional service, but a fundamental right of every child that must be fulfilled by the state. ^4^

Among the countries committed to inclusive education are Indonesia and Oman. Indonesia, as a major country in Southeast Asia, has a progressive regulatory framework for inclusive education. This is reflected in the Minister of National Education Regulation No. 70 of 2009 concerning Inclusive Education, as well as its strengthening thru Law No. 8 of 2016 ^5^ concerning Persons with Disabilities. However, its implementation still faces challenges such as limited teacher competence, school unpreparedness, and social stigma toward children with special needs. ^6– 9^

Meanwhile, Oman, a small country in the Middle East region with peaceful and simple social characteristics, presents a different dynamic. This country adopted formal inclusive policies and international initiatives relatively early, including Child-Friendly Education (CFE) in collaboration with UNICEF. ^10^ Additionally, Oman has a long-term development direction thru Oman Vision 2040, which emphasizes fair, innovative, and inclusive education. ^11^ From a cultural perspective, Oman and Indonesia both share Eastern values and moderate Islam, which serve as the foundation for building social and educational harmony. ^12^ This context makes Oman and Indonesia interesting to compare, especially since both manage cultural diversity while also striving to respond to global demands for inclusive education.

Furthermore, fundamental differences in educational governance add another important reason for conducting this comparative study. Indonesia implements a decentralized system, where local governments have significant authority in education administration, including inclusive education. Conversely, Oman adopts a more centralized system, with education policies directly controlled by the central government. Although this was not the focus of the research, these differences in governance patterns have implications for variations in implementation in the field, both in terms of planning, community support, and teacher readiness.

Previous research on inclusive education has generally focused on the experiences of a single country or even a single school. ^13, 14^ Moreover, Cross-country studies, particularly those comparing Southeast Asia and the Middle East, are still limited. Additionally, while many studies highlight regulatory or policy aspects, few have connected global frameworks like Universal Design for Learning (UDL) and Individualized Education Programs (IEPs) with real-world classroom practices. Therefore, according to the researchers, this condition is important to examine, as there are often significant differences between ambitious regulations and everyday learning practices.

Additionally, cross-national research on inclusive education has largely been conducted involving Western countries, such as the United States, Canada, or European nations. ^15– 17^ These studies do enrich the literature, but the cultural, social, and religious contexts of Western countries are vastly different from those of Indonesia or Oman. Therefore, a comparative study between countries that share both Eastern culture and moderate Islam is important to enrich a more diverse global perspective. ^18^

Therefore, this study aims to analyze the implementation of inclusive education in Oman and Indonesia using a comparative approach. The focus is on uncovering the similarities and differences in the practice of inclusive education in the field. Hence, this research is expected not only to enrich the academic discourse on inclusive education but also to provide practical contributions for policymakers, educational leaders, and practitioners.

### Literature review

The implementation of inclusive education, which emphasizes that regular schools with an inclusive orientation are the most effective way to combat discrimination, build a friendly society, and improve the quality of education for all children, has become a global consensus. This agreement is outlined in the document The Salamanca Statement. ^1^ This document became the foundation for the inclusion movement worldwide and shifted the educational paradigm from segregation toward diversity as a strength. Furthermore, the United Nations Convention on the Rights of Persons with Disabilities ^3^ affirms that inclusive education is a human right for all children in the world. All countries that have ratified this convention, including Oman and Indonesia, have a legal and moral obligation to ensure equal education for children with special needs and eliminate the barriers they face. Thus, inclusion is not only understood as a policy choice, but as a global mandate that must be translated into national educational practices.

In line with this mandate, the theory and implementation framework for inclusion emphasize that every child has the potential to learn. Florian and Black-Hawkins, ^15^ thru their theory of inclusive pedagogy, emphasize the importance of teacher flexibility in adapting learning strategies, rather than forcing students to conform to a rigid system. The Universal Design for Learning (UDL) framework reinforces this principle by promoting curriculum flexibility thru diverse ways of representing, motivating, and expressing learning outcomes. ^19^ On a practical level, implementing inclusion requires an Individualized Education Program (IEP) or Individualized Learning Program (PPI: *Program pembelajaran individual* ), which provides a measurable learning plan tailored to each child’s needs. Moreover, Gibson and Blandford, ^20^ emphasized the important role of the Special Educational Needs Coordinator (SENCO) in the UK to ensure the IEP is implemented, while in Indonesia this role is carried out by the Special Guidance Teacher. ^21^ Thus, personalized learning becomes the core of inclusion implementation so that every student can develop their maximum potential.

In addition, the implementation of inclusive education also requires clear and integrated service models. The service model emphasizes the importance of collaboration between school elements, experts (such as therapists or psychologists), and the community thru multidisciplinary support teams. On the other hand, Gibson and Blandford ^20^ emphasize that cross-sectoral coordination is key to assessing student needs, designing accommodations, and providing ongoing support. Additionally, participatory school management is essential for building an inclusive culture. Kozikoglu and Uca ^22^ emphasize the role of the principal in fostering collaboration among teachers, students, and parents to create a school climate that is welcoming to diversity. Without strong team support and a solid service model, inclusion efforts are vulnerable to being partial and unsustainable.

Furthemore, teacher readiness thru training is also a determining factor for the success of inclusion. Pre-service and in-service training programs must emphasize differentiated pedagogical strategies, inclusive classroom management, the use of the UDL framework, and the development and implementation of IEPs. Research shows that teaching and training experience influences the effectiveness of inclusion practices. Emam and Al-Mahdy ^14^ found that in Oman, gender and teacher experience significantly influenced inclusive practices, while Phytanza et al. ^23^ in Indonesia confirmed that limited teacher training and the absence of standardized IEPs were the main obstacles. This means that without continuous training programs, inclusion policies will only remain at the formal level without real transformation in the classroom.

Another crucial aspect is curriculum adaptation and instructional accommodations. The principle of curriculum diversification allows for the presentation of varied material according to student needs, including language simplification, the provision of alternative materials, and the use of assistive technology. The Indonesian Ministry of Education and Culture ^24^ emphasizes that curriculum adaptation and multi-level support from the government, schools, community, and parents are necessary for inclusive learning to be effective. If this is ignored, students with different needs are at risk of falling behind academically and widening the education gap.

Additionally, community involvement, parental support, and support networks are crucial factors for the sustainability of inclusive education. Gibson and Blandford ^20^ showed that parental and community participation is not merely an additional support, but rather an integral part of the inclusive service system. Community support strengthens school resources and helps create a cohesive learning environment. However, cultural factors often pose an obstacle. Mukhlis ^6^ notes that despite Indonesia having a clear legal framework, social stigma against disability remains strong. Meanwhile, in Oman, the centralized system facilitates policy coordination, but community participation is often minimal. ^25^ This shows that both centralistic and decentralistic models face their own challenges in implementing inclusion.

Moreover, the literature also confirms that failure to implement inclusion theories has serious consequences. Children with special needs may face academic and social marginalization, while teachers are locked in uniform approaches that disregard difference, and formal policies lose their practical usefulness. On the structural level, highly centralized administration encourages low community participation, whereas a decentralized system has the potential to produce inter-regional inequities due to differences in teacher capacity and resources. ^26^ Thus, the realization of inclusion necessitates a balance between adaptive and participative centralization and uniform and equal decentralization.

Overall, this literature review confirms that effective implementation of inclusive education requires a synergistic interaction between global normative foundations, inclusive pedagogy theories, teacher training, personalization of learning through IEPs/PPIs according to children’s needs, curriculum adaptation, community involvement, and cultural attitude transformation. Considering all these elements, an inclusive education system can move from mere policy formality to real practice that empowers all children to learn fairly, appropriately, and with dignity.

## Method

This study employed document analysis as described by Bowen, ^27^ which involved scanning, reading, and interpreting policy documents and implementation reports on inclusive education, conducted from May 2025 to August 2025. The document sources included twenty-seven national regulations, technical guidelines, and field implementation reports. The Indonesian documents were obtained through the Ministry of Education and Culture, ^24^ while the Oman documents ^28– 30^ were obtained directly through the study’s co-author, a Professor and Director of the Faculty of Sharia at the Omani Ministry of Education, who is also a co-author of this article. This strengthened the validity of the sources by providing access to official and up-to-date data on the implementation of inclusive education in Oman.

The 33 documents included policy documents, guidelines, and implementation documents, as well as peer-reviewed research manuscripts, which were reviewed for context and depth. The documents were then reviewed and analyzed across five main dimensions: 1) Service Models, Support Teams, and School Management; 2) Teacher Training; 3) Curriculum Adaptation and Learning Accommodations; 4) Community, Parent, and Support Networks; and 5) Cultural Attitudes. Information from academic research, international frameworks, and national policy documents was compared to ensure triangulation. This process helped communities in Oman and Indonesia understand how inclusive education is implemented effectively and equitably.

Data analysis was conducted by collecting and reviewing various policy documents and reports on the implementation of inclusive education from Indonesia and Oman through online searches using keywords: “policies and implementation of Inclusive education in Indonesia and Oman” and documents provided by co-authors in Oman. After collecting the documents, the researchers conducted in-depth readings to understand their content and context, then interpreted their meaning. The information obtained was then classified into five analytical dimensions: service models and support teams; teacher training; curriculum adaptation; community and parent involvement; and cultural attitudes. In the next stage, the researchers analyzed and synthesized the document content to identify patterns and differences, while also verifying the validity and credibility of the sources.

## Results and discussion

### 1. Service model, support teams, and school management

Document analysis shows that both Oman and Indonesia emphasize a service model oriented toward the diverse needs of students. In Oman, this approach is more centrally coordinated thru the ministry. Official documents such as Special Education Services in the Sultanate of Oman ^28^ ensuring that every school has a formal framework for identifying student needs and providing professional support. However, community participation and school flexibility are still limited due to the centralized system. ^31, 32^ Conversely, in Indonesia, the service model is more varied due to the influence of educational decentralization. Schools have the flexibility to form support teams consisting of regular teachers, special guidance teachers (GPK: *Guru Pembimbing Khusus*), school principals, and in some cases involving psychologists or experts. ^33– 35^ However, there is still a wide variation in quality between schools due to limited resources and differences in school management commitment. ^36, 37^

### 2. Teacher training

The findings indicate that teacher training is a critical factor for successful inclusion. In Oman, teacher training is systematically implemented thru a national program designed by the ministry, although research by Emam & Al-Mahdy ^14^ shows there are still gaps in teachers’ experience and perceptions of inclusion, for example: Teachers with moderate experience seem to be “lagging behind” in some dimensions of efficacy compared to new and very experienced teachers, particularly in the aspect of collaboration. Meanwhile, in Indonesia, the presence of GPK is an important instrument, but training is still uneven and depends on local government and partner support. ^38, 39^ This leads to inconsistency in teachers’ competence in implementing inclusive instructional strategies. ^23^

### 3. Curriculum adaptation and instructional accommodation

Both Oman and Indonesia recognize the importance of curriculum adaptation as an integral part of inclusion. Oman’s policy documents explicitly emphasize the alignment between the national curriculum and students’ individual needs, but limitations in implementation at the classroom level persist due to the dominance of standardized approaches. ^40^ Indonesia, thru the Ministry of Education and Culture ^24^ guidelines, encourages curriculum diversification and the implementation of Individualized Learning Programs (PPI/IEP). However, in practice, there are still obstacles due to the lack of standard IEP guidelines and limited teacher training in developing and implementing PPIs. For example, there are no uniform technical guidelines for IEPs/PPIs, and teachers, including classroom teachers and GPKs (Special Needs Assistants), have not received sufficient training to develop PPIs that meet children’s needs. ^26, 41, 42^ As a result, learning accommodations in the classroom are often inconsistent. ^43^

### 4. Community, parent, and support network

Involvement Community and parental participation are distinguishing aspects of both countries. In Oman, the centralized education system makes coordination between government levels easier, but the space for public participation is relatively limited. ^25, 31^ The government relies on official data, such as the Statistical Report on Persons with Disabilities in the Sultanate of Oman, ^29^ for strategic planning. Furthermore, the commitment to improving services is outlined in policy documents like Inclusive Education Services for Persons with Disabilities in Oman, ^30^ which serves as a guide for multi-sectoral support. Conversely, in Indonesia, the existence of a culture of mutual cooperation and the role of local communities provides greater opportunities for community participation. ^33, 34^ However, the level of engagement still varies greatly between regions, depending on school initiatives and local government support. Parental involvement in Indonesia is also still hindered by low levels of inclusive education literacy, often remaining purely administrative. ^39^

### 5. Cultural attitudes and stigma

Cultural attitudes and stigma remain a serious challenge to the implementation of inclusive education. In Indonesia, research by Mukhlis ^6^ shows that although the legal framework for inclusion is clear, social stigma against people with disabilities is still strong, both among students and parents. This puts children with special needs at risk of discrimination and marginalization in regular schools. In Oman, although society is known to be moderate, ^12, 31^ public participation in supporting inclusion remains low because the system’s orientation places more emphasis on top-down policies. As a result, public awareness of the importance of inclusive education has not yet fully taken root at the community level. ^14^

Table 1 below shows a comparative overview of inclusive education implementation in Indonesia and Oman.

**Table 1.** Comparative overview of inclusive education in Oman and Indonesia.

Dimensions	Indonesia	Oman
**Service Model, Support Teams, & School Management**	Decentralization provides flexibility for schools to form support teams (special education teachers, regular teachers, principals, psychologists). However, the quality varies due to limited resources and the commitment of school management.	A centralized system provides a uniform formal framework for identifying student needs. However, community participation and school flexibility are limited.
**Teacher Training**	GPK is the main instrument, but training is uneven, heavily reliant on local government and partner support. Inclusion teacher competency is inconsistent.	Training is conducted thru a national program, making it more systematic. However, a study ^14^ shows there are still gaps in teachers' experiences and perceptions.
**Curriculum Adaptation & Instructional Accommodation**	Adaptive curricula are recommended, ^24^ including PPI/IEPs, but implementation is weak due to a lack of standards and teacher training. Class adaptation is inconsistent.	The policy document emphasizes adapting the curriculum to students' needs, but practice is still hampered by the dominance of the national standard approach.
**Community, Parent, & Support Network**	Gotong royong provides a broader opportunity for community participation. However, parental involvement is still low and tends to be administrative.	Coordination is easier because of the centralized system, but the space for public participation is relatively limited. ^25^
**Cultural Attitudes & Stigma**	Disability stigma is still strong in society, ^6^ leading to discrimination and marginalization.	The society is moderate, ^12^ but public awareness is low due to the top-down approach.

As shown in Table 1, there are significant differences in the implementation of inclusive education between Indonesia and Oman, especially regarding governance, community participation, and teacher training.

Based on Table 1, the research findings indicate that both Indonesia and Oman face challenges in implementing inclusive education, although within different governance contexts. The difference between centralized and decentralized models affects service patterns, community involvement, and implementation consistency.

In Indonesia, decentralization enables innovation at the school level and opens up space for community participation. However, this condition creates disparities between regions. Some schools excel at building multidisciplinary support teams, while others still lack resources. This finding aligns with Phytanza et al., ^23^ who confirmed that the absence of IEP standards leads to highly variable quality of implementation.

Meanwhile, Oman benefits from strong coordination thru a centralized system. Every school has a clear framework for inclusion services. However, the top-down approach limits the space for public participation. ADB ^25^ and Emam ^44^ studies confirm that formal policies are not always rooted in everyday practices, especially when it comes to involving families and communities.

From the perspective of teacher training, both countries face capacity constraints. Oman has a more established national system, but gaps in teacher experience are still evident. ^14^ Indonesia has GPK as an important element, but the distribution and quality of training are uneven. This strengthens the argument that successful inclusion requires ongoing, practice-based training, not just formal policies. ^45^

Curriculum adaptation is also a weak point. Indonesia has already regulated this in the Ministry of Education and Culture ^24^ guidelines, but in practice, it is often hampered by limitations in teacher competence. Oman also faces similar obstacles due to the dominance of the standard curriculum. This confirms the urgency of implementing the UDL framework ^19^ and IEP to bridge regulations and classroom practices.

Cultural factors present fundamental challenges. Indonesia still faces stereotypes and social discrimination against individuals with disabilities. ^6^ Meanwhile, in Oman, public awareness remains low due to a policy approach that is too government-centric. Both conditions indicate that cultural transformation is a prerequisite for the sustainability of inclusion. Without a change in public attitude, formal policies will continue to face resistance in practice.

Overall, the findings of this study demonstrate that the implementation of inclusive education in Oman and Indonesia is shaped by contrasting governance models, each with its advantages and constraints. Oman’s centralized system enables strong coordination and consistency, yet it restricts opportunities for broad public participation. Indonesia’s decentralized model, on the other hand, provides more space for community involvement but struggles with uneven quality and disparities across regions. This comparative perspective underscores that inclusive education cannot be secured through governance design alone; it requires an integrated effort that combines robust national policies, sustained teacher training, flexible curriculum adaptation, multidisciplinary team support, and a cultural transformation that normalizes inclusivity within society. Building on this insight, the study advances a novel proposition: a hybrid model that blends adaptive centralization with equitable decentralization, offering a balanced strategy to ensure inclusive education operates effectively and sustainably across diverse contexts.

Based on the discussion above, the strength of this study lies in weaving global frameworks such as UDL and IEP into a rarely studied cross-country comparative analysis, revealing how contrasting governance logics—centralization in Oman and decentralization in Indonesia—shape the way international commitments are translated into everyday learning practices. Beyond advancing scholarly debate, this research offers practical value by offering concrete recommendations to policymakers, educational leaders, and practitioners seeking to embed inclusive principles more deeply into their systems. The recommendations presented here aim to guide both countries in strengthening inclusive practices while also contributing to the broader global effort to ensure that education is recognized and realized as a right for every child in every context.

## Conclusion

The results of this study indicate that the implementation of inclusive education in Indonesia and Oman operates with different dynamics depending on the adopted education governance system. Indonesia, with its decentralization model, provides room for flexibility and innovation at the school level, as well as significant opportunities for community participation. However, this condition also creates disparities between regions due to differences in teacher capacity, resources, and school management commitment. Conversely, Oman, which implements a centralized system, is able to ensure policy consistency and stronger national coordination, but community involvement and school flexibility are limited. Both countries still face similar challenges, including limited teacher training, weak curriculum adaptation, and strong cultural stigma against disabilities. Overall, this study confirms that effective inclusive education can only be achieved thru a combination of clear national policies, ongoing teacher training, a flexible UDL and IEP-based curriculum, active community involvement, and a more inclusive cultural transformation.

### Recommendations

Based on these findings, this study recommends that governments in both countries strengthen regulations by developing national IEP or PPI standards that can be adapted to the local context, while also ensuring adequate allocation of resources and infrastructure for inclusive schools. At the school level, teachers need to receive continuous capacity development with a focus on differentiated learning strategies, IEP development, and the implementation of the UDL framework. Collaboration in the form of multidisciplinary support teams involving regular teachers, special education teachers, professionals, and parents must be continuously improved. Additionally, the community and parents also need to be provided with inclusive education literacy so that their role doesn’t stop at the administrative aspect, but extends to active participation in the learning and decision-making process. With this strategy, both countries are expected to strengthen the integration of the principle of inclusion so that education truly becomes a right accessible to all children.

### Limitations & Further direction

This study has a number of limitations. First, the analysis is based solely on policy documents, implementation reports, and academic literature, so it does not fully capture the reality of practices on the ground. Second, data from Oman is largely sourced from official government documents, so the potential for top-down bias remains. Third, this research focuses on five main dimensions: service model, teacher training, curriculum adaptation, community involvement, and cultural attitudes, so other factors such as the role of digital technology in supporting inclusion or the direct perspectives of students have not been revealed. Fourth, the generalization of research findings is also limited because the social and cultural contexts of Indonesia and Oman have unique characteristics that may not be fully comparable to other countries. Therefore, further research needs to involve more in-depth field data, obtained thru interviews, observations, and case studies, in order to provide a more comprehensive picture of inclusion implementation.

## Data Availability

No data are associated with this article. (No data was uploaded due to secondary data as explained in the methodology section). Figshare: Comparative Dataset of Inclusive Education Policy and Implementation in Indonesia and Oman. **https://doi.org/10.6084/m9.figshare.30314296.v1** . ^46^ The project contains the following underlying data:
•
Inclusive_Education_Data_Indonesia_Oman_fix.docx Data are available under the terms of the Creative Commons Attribution 4.0 International license (CC-BY 4.0).
