# Supplementary material for: Inclusive Education Implementation in Oman and Indonesia: A Comparative Analysis Study
Source: F1000Res. 2025 Dec 31;14:1361. Originally published 2025 Dec 5. [Version 2] doi: 10.12688/f1000research.171420.2 (PMC12809024; doi:10.12688/f1000research.171420.2)
Supplement: Peer review history

# Reviewer report: Reviewer response for version 2

**DOI:** 10.5256/f1000research.194348.r446967

**Published:** 2026-01-15

**Authors:** Reza Rachmadtullah

**Affiliations:** 1 PGRI Adi Buana Surabaya University, Surabaya, Indonesia

This article makes a significant contribution to the study of inclusive education through a comparative approach between Indonesia and Oman, two non-Western countries rarely studied side-by-side. The article's primary strength lies in its successful linking of socio-cultural contexts with educational governance structures to explain variations in the implementation of inclusive education. The author's use of qualitative policy document analysis of 33 official documents allows the authors to present a robust systemic picture of the practices, challenges, and direction of inclusion policies in both countries.

 The results demonstrate a sharp and balanced analysis. Oman's centralized education system ensures policy consistency but lacks adaptation to local needs and community participation. Conversely, decentralization in Indonesia encourages local innovation and community involvement but creates disparities in the quality of implementation across regions. These findings enrich the global discourse by emphasizing that no single governance model is entirely ideal for inclusive education; rather, a contextually tailored, hybrid approach is needed.

 The article also consistently identifies cross-contextual issues, such as limited teacher preparedness, weak curriculum adaptation, and strong cultural stigma against disabilities. This discussion is relevant and aligned with recent literature, particularly in relation to Universal Design for Learning (UDL) and the Individualized Education Program (IEP). However, the article could be strengthened by incorporating an implementation perspective at the school level or the voices of practitioners (teachers and principals) to complement the document-based analysis.

 Overall, this article is academically sound, policy-relevant, and offers applicable recommendations for developing countries with similar social and cultural characteristics. This study is a worthy reference in developing inclusive education policies, particularly for policymakers, comparative education researchers, and practitioners seeking to bridge the gap between regulations and inclusive practices on the ground.

Is the work clearly and accurately presented and does it cite the current literature?

Yes

If applicable, is the statistical analysis and its interpretation appropriate?

Yes

Are all the source data underlying the results available to ensure full reproducibility?

Yes

Is the study design appropriate and is the work technically sound?

Yes

Are the conclusions drawn adequately supported by the results?

Yes

Are sufficient details of methods and analysis provided to allow replication by others?

Yes

Reviewer Expertise:

Focus on research in the field of education, research and educational research methods

I confirm that I have read this submission and believe that I have an appropriate level of expertise to confirm that it is of an acceptable scientific standard.

# Reviewer report: Reviewer response for version 2

**DOI:** 10.5256/f1000research.194348.r446366

**Published:** 2026-01-07

**Authors:** Rasmitadila Rasmitadila

**Affiliations:** 1 Universitas Djuanda, Bogor, Indonesia

Dear Authors,

 Thank you for your revision to increase the quality of the manuscript. Your revisions are very detail and suitable for the publication. 

 Best regards,

 Rasmitadila

Is the work clearly and accurately presented and does it cite the current literature?

Yes

If applicable, is the statistical analysis and its interpretation appropriate?

Partly

Are all the source data underlying the results available to ensure full reproducibility?

Yes

Is the study design appropriate and is the work technically sound?

Yes

Are the conclusions drawn adequately supported by the results?

Yes

Are sufficient details of methods and analysis provided to allow replication by others?

Partly

Reviewer Expertise:

Inclusive education in Elementary School

I confirm that I have read this submission and believe that I have an appropriate level of expertise to confirm that it is of an acceptable scientific standard.

# Reviewer report: Reviewer response for version 1

**DOI:** 10.5256/f1000research.189028.r439556

**Published:** 2025-12-16

**Authors:** Rasmitadila Rasmitadila

**Affiliations:** 1 Universitas Djuanda, Bogor, Indonesia

Strengths:

 The research gap is clearly and strongly identified from the abstract to the introduction. The author uses a global theoretical framework connecting Universal Design for Learning (UDL); IEP/PPI; and Inclusive Pedagogy. The comparison between Oman and Indonesia is consistent across five dimensions: service models, teacher training, curriculum adaptation, community involvement, and cultural attitudes.

 Suggestions:

 The author could add a critique of the existing literature to justify the need for this comparative study and identify specific gaps that have not been addressed by previous research.

 The analysis process does not yet provide the procedural details required by qualitative research standards. The author could explain the inclusion and exclusion criteria for documents, how to determine source credibility, and the analysis steps used, such as coding, categorization, or triangulation techniques, to enhance methodological transparency. It would be beneficial to include a document analysis flowchart and academic rationale for selecting the five dimensions of analysis to enhance the robustness of the method.

Is the work clearly and accurately presented and does it cite the current literature?

Yes

If applicable, is the statistical analysis and its interpretation appropriate?

Partly

Are all the source data underlying the results available to ensure full reproducibility?

Yes

Is the study design appropriate and is the work technically sound?

Yes

Are the conclusions drawn adequately supported by the results?

Yes

Are sufficient details of methods and analysis provided to allow replication by others?

Partly

Reviewer Expertise:

Inclusive education in Elementary School

I confirm that I have read this submission and believe that I have an appropriate level of expertise to confirm that it is of an acceptable scientific standard, however I have significant reservations, as outlined above.

# Response

**Published:** 2025-12-20

**Authors:** Siti Masyithoh

**Affiliations:** PGMI, Universitas Islam Negeri Syarif Hidayatullah Jakarta, South Tangerang, Banten, Indonesia

Dear Ms. Rasmitadila,

 We sincerely thank the reviewers for their careful reading of our manuscript and for their insightful, constructive, and encouraging comments. We greatly appreciate the recognition of the strengths of this research, particularly regarding the clarity of the research gap, the coherence of the comparative framework, and the integration of global theoretical perspectives such as Universal Design for Learning (UDL), Individualized Education Programs (IEP/IPC), and Inclusive Pedagogy.

 We have carefully addressed all suggestions made by the reviewers. Specifically, we have strengthened the manuscript in the following ways:

 1. Critical Engagement with the Literature

 We revised the final section of the introduction to provide a more explicit critique of existing studies. This includes clearer identification of unresolved gaps in previous research, particularly the limited number of cross-regional comparative studies between Southeast Asia and the Middle East and the lack of connections between global inclusive education frameworks and everyday implementation practices in non-Western contexts.

 2. Methodological Transparency and Rigor

 The Methods section has been substantially expanded to clarify the qualitative document analysis procedures. We now explicitly explain the inclusion and exclusion criteria for document selection, the strategies used to assess source credibility, and the analytical steps employed, including categorization, thematic comparison, and triangulation across policy documents, implementation reports, and academic literature. Furthermore, we have strengthened the academic basis for selecting the five analytical dimensions by directly linking them to the reviewed theory and previous empirical findings.

 3. Clarity and Replicability

 Revisions were also made to improve clarity, coherence, and transparency throughout the manuscript to support interpretive validity and facilitate replication by other researchers.

 We believe that these revisions have significantly improved the robustness, clarity, and scientific contribution of the manuscript. We are grateful for the valuable guidance from the reviewers, who have helped refine the conceptual and methodological quality of this study.

 Thank you for the opportunity to revise and resubmit our work. We hope this revised manuscript meets the journal's standards and look forward to your further consideration.

 Sincerely,

 Siti Masyithoh

 (one of the authors)
